# Outbreak among drug users caused by a clonal strain of group A streptococcus.

**DOI:** 10.3201/eid0602.000211

**Published:** 2000

**Authors:** L M Böhlen, K Mühlemann, O Dubuis, C Aebi, M G Täuber

**Affiliations:** Institute of Medical Microbiology, Berne, Switzerland.

## Abstract

We describe an outbreak among drug users of severe soft-tissue infections caused by a clonal strain of group A streptococcus of M-type 25. Cases (n = 19) in drug users were defined as infections (mainly needle abscesses) due to the outbreak strain. Comparison with controls showed that infected drug users bought drugs more often at a specific place. Drug purchase and use habits may have contributed to this outbreak.


### Outbreak among Drug Users Caused by a Clonal Strain of Group A Streptococcus

**Lorenz M. Böhlen, Kathrin Mühlemann, Olivier Dubuis, Christoph Aebi, and Martin G. Täuber**
University of Berne, Berne, Switzerland

We describe an outbreak among drug users of severe soft-tissue infections caused by a clonal strain of group A streptococcus of M-type 25. Cases (n = 19) in drug users were defined as infections (mainly needle abscesses) due to the outbreak strain. Comparison with controls showed that infected drug users bought drugs more often at a specific place. Drug purchase and use habits may have contributed to this outbreak.

In the 1980s, reemergence of severe group A streptococcus (GAS) infections, especially toxic shock syndrome, necrotizing fasciitis, and bacteremia associated with high death rates, was observed (1,2). Temporal and geographic clustering of cases with severe GAS infection has been described (3,4). Outbreaks caused by clonal strains have been reported in households, schools, and hospitals (5).

In September 1997, a sudden increase was observed in needle abscesses due to GAS among drug users hospitalized in Berne, Switzerland. Analysis of GAS isolates suggested that the outbreak was caused by clonal strains. An outbreak investigation included a case-control study of potential sources and risk factors for acquisition of GAS infection among drug users in Berne. To our knowledge, this is the first report of an outbreak among drug users of invasive GAS infections caused by clonal strains.

### The Study

Cases of GAS infection were identified through culture records of the Institute of Medical Microbiology, University of Berne, Switzerland. During September to December 1997, all GAS isolates from any site were prospectively stored and included in the study. Cases were defined as GAS infection in drug users from September 22 to November 20, 1997, due to the outbreak strain, as determined by pulsed-field gel electrophoresis (PFGE).

During December 15-20, 1997, controls were randomly chosen from drug users visiting a government shelter for drug use and needle exchange in Berne. An exclusion criterion was a skin infection at an injection site since September.

Drug users with cases, as well as controls, were interviewed by a standardized questionnaire including age, employment, recent infections, and drug use habits (Table). Crude odds ratios and 95% confidence intervals were calculated with EpiInfo software, version 6.02 (CDC, Atlanta, GA). Categorical data were compared with Fisher's exact test or the chi-square test, and continuous data with Student's t-test. To identify independent risk factors, logistic regression analysis was performed in EGRET (Seattle, WA).

GAS was isolated on sheep blood agar and identified by gram staining and the bacitracin test (6). Throat swabs were obtained at the time of the interview from the 55 controls. Culture specimens were taken with moistened (sterile saline) swabs from spoons (n = 10) and filters (n = 2) used for drug preparation.

Samples of cocaine confiscated by police during September 15-28 and October 12-20 were cultured for GAS. The drug specimens had been stored for 1 to 6 weeks in a dry place before culture. For each cocaine specimen, three 0.2-g samples were dissolved in sterile saline. One sample was inoculated into tryptic soy broth (TSB); the other two were filtered through sterile 0.45-µm membranes. One filter was placed on a sheep blood agar plate, and the other was

Address for correspondence: Kathrin Mühlemann, Institute of Medical Microbiology, Friedbühlstrasse 51, CH-3010 Berne, Switzerland; fax: 41-31-632-3550; e-mail: muehlemann@imm.unibe.ch.

Table. Sociodemographic and drug use habits of 19 drug users with group A streptococcal infection and 55 healthy controls, Berne, Switzerland, 1997

| Variable | Cases n=19 N | % | Controls n=55 N | % | Odds Ratio | 95% CI[a] |
|---|---|---|---|---|---|---|
| Age | | | | | | |
| Mean (yrs) | 30.3 | 30.7 | | | | |
| Range (yrs) | 20-43 | 21-48 | | | | |
| Sex | | | | | | |
| Male | 9 | 47.4 | 38 | 69.1 | Ref. | 0.76-8.26 |
| Female | 10 | 52.6 | 17 | 30.9 | 2.48 | |
| Employed | | | | | | |
| No | 12 | 63.2 | 33 | 60.0 | Ref. | |
| Yes | 6 | 31.6 | 15 | 27.3 | 1.10 | 0.30-4.00 |
| Unknown | 1 | 5.3 | 7 | 12.7 | | |
| Daytime shelter | | | | | | |
| Home or work | 7 | 36.9 | 20 | 36.4 | Ref. | |
| Street | 8 | 42.1 | 20 | 36.4 | 1.14 | 0.30-4.40 |
| Unknown | 4 | 21.1 | 15 | 27.2 | | |
| Nighttime shelter | | | | | | |
| Home | 14 | 73.7 | 29 | 52.7 | Ref. | |
| Street | 3 | 15.8 | 10 | 18.2 | 0.62 | 0.10-3.06 |
| Unknown | 2 | 10.5 | 16 | 29.1 | | |
| Sharing of paraphernalia for drug use | | | | | | |
| Filter | | | | | | |
| No | 9 | 47.4 | 34 | 61.8 | Ref. | |
| Yes | 8 | 42.1 | 21 | 38.2 | 1.44 | 0.42-4.92 |
| Unknown | 2 | 10.5 | 0 | 0.0 | | |
| Spoon | | | | | | |
| No | 8 | 42.1 | 28 | 50.9 | Ref. | |
| Yes | 9 | 47.4 | 27 | 49.1 | 1.08 | 0.32-3.68 |
| Unknown | 2 | 10.5 | 0 | 0.0 | | |
| Needle | | | | | | |
| No | 15 | 78.9 | 49 | 89.1 | Ref. | |
| Yes | 1 | 5.3 | 5 | 9.1 | 0.65 | 0.03-6.68 |
| Unknown | 3 | 15.8 | 1 | 1.8 | | |
| Cocaine heating | | | | | | |
| No | 16 | 84.2 | 35 | 63.6 | | |
| Yes | 0 | 0.0 | 6 | 10.9 | p = 0.17 | |
| Unknown | 3 | 15.8 | 14 | 25.5 | | |
| Mode of drug use | | | | | | |
| Intravenous[b] | 17 | 89.3 | 55 | 100.0 | | |
| Mucosal only | 1 | 5.3 | 0 | 0.0 | | |
| Unknown | 1 | 5.3 | 0 | 0.0 | | |
| Illegal drug used | | | | | | |
| Cocaine | 2 | 10.5 | 7 | 127 | | |
| Heroin | 0 | 0.0 | 2 | 3.6 | | |
| Both | 16 | 84.2 | 46 | 83.6 | | |
| Unknown | 1 | 5.3 | 0 | 0.0 | | |
| Methadone program[c] | | | | | | |
| No | 18 | 94.7 | 40 | 72.7 | Ref. | |
| Yes | 1 | 5.3 | 15 | 27.3 | 0.15 | 0.01-1.23 |
| No. of drug use(s) | | | | | | |
| ≤1 per day | 4 | 21.1 | 21 | 38.2 | Ref. | |
| >1 per day | 13 | 68.4 | 29 | 52.7 | 2.35 | 0.59-10.04 |
| Unknown | 2 | 10.5 | 5 | 9.1 | | |
| Cocaine used | | | | | | |
| ≤1 g per day | 7 | 36.8 | 30 | 54.5 | Ref. | |
| >1 g per day | 8 | 42.1 | 10 | 18.2 | 3.43 | 0.84-14.34 |
| Unknown | 4 | 21.1 | 15 | 27.3 | | |
| Nationality of dealer(s) | | | | | | |
| Not A | 4 | 21.1 | 15 | 27.3 | Ref. | |
| Only A | 6 | 31.6 | 13 | 23.6 | 1.73[d] | 0.32-9.59 |
| Several including A | 4 | 21.1 | 20 | 36.4 | | |
| Unknown | 5 | 26.3 | 71 | 2.7 | | |
| Dealer location | | | | | | |
| Place X | 8 | 42.1 | 1 | 1.8 | 27.43 | 2.57-696.75 |
| Other places | 7 | 36.8 | 24 | 43.6 | Ref. | p <0.001 |
| Not Berne | 0 | 0.0 | 1 | 1.8 | | |
| Unknown | 4 | 21.1 | 29 | 52.7 | | |

[a]95% confidence interval
[b]Includes combined intravenous and mucosal drug use for one case and seven controls.
[c]Government-approved methadone program.
[d]Comparing "only nationality A" and "not A."

inoculated into 500 ml TSB. Cultures were incubated at 37°C. Subcultures from TSB were placed on sheep blood agar at 12-hour intervals until the broth became turbid.

PFGE was performed on GAS isolates as described (7). Briefly, whole genomic DNA was restricted with SmaI, and fragments were separated in a CHEF DRIII unit (BioRad, Glattbrugg, Switzerland) under the following conditions: 0.5xTRIS-borate-EDTA running buffer, 6 volts/cm, 14°C, 120°C angle, and a 1.2- to 54-s ramped switch time for 18 hours. Gels were stained with bromide, and banding patterns were compared. Only isolates with identical banding patterns were considered to belong to the same clone.

The M-type of the outbreak strain was determined by PCR amplification and sequencing of a region of the emm gene (3,8). The presence of the pyrogenic exotoxin A gene was evaluated as described (3).

PFGE analysis of isolates showed that 19 of the 21 infections were caused by the same clone (Figure). Most of these infections (16 of 19) were needle abscesses at the injection site; two were complicated by erysipelas and one by osteomyelitis distant from the needle abscess. None of the patients had streptococcal toxic shock syndrome (1). Seventeen cases required inpatient treatment, including surgery; all patients recovered. All patients lived (n = 16) or purchased drugs in Berne (n = 3).

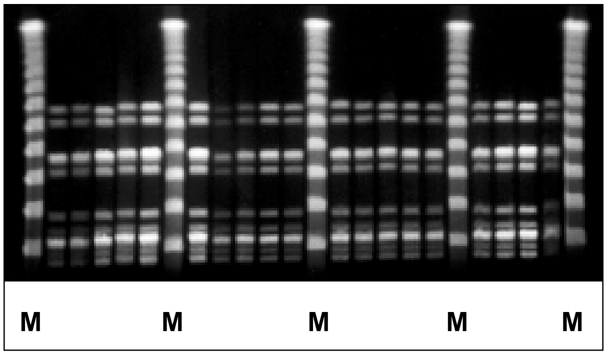

Figure. Pulsed-field gel electrophoresis of group A streptococci causing clinical infection in 19 drug users, Berne, Switzerland.
M = molecular weight standards. All other lanes represent PFGE pattern of group A streptococcus isolates from 19 drug users.

Cases and controls did not differ by mean age, current employment status, sharing of paraphernalia (e.g., needles, spoons, and filters) for drug use, and place of shelter (Table).

No GAS could be isolated from the eight confiscated cocaine samples. Five (9%) of 55 controls were colonized by GAS at the time of the interview; three of them carried the outbreak strain. Unfortunately, four of the five colonized controls refused to disclose the location and nationality of their drug dealer(s). One admitted buying drugs in various places, including place X, and from dealers of several nationalities, including nationality A. He was colonized by the outbreak strain.

The outbreak strain was M-type 25, which did not carry the gene encoding for the pyrogenic exotoxin A. The strain was susceptible to penicillin, clindamycin, and erythromycin, as determined by E-testing.

### Conclusions

During the past decade, GAS infections have attracted increased attention because of their worldwide reemergence. Molecular typing studies suggested that this might have been due to the intercontinental spread of a virulent clone of M-type 1 (3). Isolates of other M-types have since been associated with clusters of severe infections (9). Person-to-person spread by respiratory droplets from colonized patients or asymptomatic carriers has been thought to be the main mode of transmission. Vaginal carriage of GAS in health-care workers has been associated with nosocomial spread of GAS (10). Acquisition of GAS by contaminated food and foodborne GAS outbreaks has also been described (11).

The epidemic of GAS infections among drug users described here is to our knowledge the first report of a clonal epidemic with GAS in this patient population. GAS is commonly isolated from soft-tissue infections in intravenous drug users (12,13). High rates of pharyngeal carriage with epidemic clones may play a role in this high frequency of GAS infections, although no clonality of such GAS isolates was found in two studies (14,15).

The case-control study showed a strong association between infection and purchase of drugs at X, a place commonly used for drug dealing in Berne. The association with place X

was strong enough that bias due to the high refusal rate among controls is unlikely.

Outbreaks of infections with other pathogens were previously described in drug users (e.g., *C. tetani, C. botulinum, Candida albicans*, and hepatitis A virus) (16-20). These outbreaks could be due to contamination of the drug or drug paraphernalia. In our population, sharing paraphernalia such as needles, spoons, and filters was reported infrequently and did not differ between cases and controls. We believe that this strain of GAS was spread through cocaine or its containers. Drug dealers and users often hide cocaine in their mouths during police raids. Therefore, GAS may be spread to drug users by contamination of the plastic bags containing the cocaine or the cocaine itself from persons with GAS colonization of the mouth and throat. The strong association of the outbreak with a common place of drug purchase suggests that one or several drug dealers there were colonized by the outbreak strain and spread it by contaminated drug containers or respiratory droplets. Infected drug users may also have become colonized by hiding the drug containers in their mouths. Alternatively, GAS may have entered the subcutaneous tissue directly by contaminated drug or handling paraphernalia with contaminated hands. Although we were not able to culture GAS from cocaine samples, the drug cannot be ruled out as a potential vehicle of GAS, since these samples had been stored for some weeks and GAS may not survive in sufficient numbers in dry (cocaine) powder.

We obtained a throat culture from only one of the infected drug users, because they had already received antibiotics for at least 1-2 days when they were identified by the microbiologic studies. The single throat culture showed carriage of the outbreak strain in the throat as well as at the site of infection (erysipelas of the leg). Oropharyngeal carriage of the outbreak strain was also found in 5.4% of our controls; the strain was not found in randomly selected isolates from nondrug users, indicating that the outbreak strain was probably circulating mainly in the drug user population.

Needle abscesses in drug users have been associated with cocaine use. The local vasoconstriction induced by cocaine may predispose to abscess formation (13,21). Cocaine, unlike heroin, is usually not heated before injection, since it is thought to lose its activity when heated. Failure to heat the drug likely increases the risk of inoculating pathogens. In our study, none of the 19 cases heated the dissolved cocaine, while six of the controls did. Some reported that they started to heat the drug after hearing about the outbreak.

From the end of the outbreak in November 1997 to May 1998, we observed three sporadic cases of infection due to the outbreak strain among drug users, but not the general population. A retrospective analysis by PFGE of clinical GAS isolates cultured in our institution demonstrated that the outbreak strain has been circulating among drug users in Berne since at least February 1997. This study also revealed two previous clonal GAS outbreaks among drug users in 1993 (unpubl. obs.). These findings suggest that GAS outbreaks may be observed among drug users more frequently than previously appreciated and that their propagation may involve transmission of the outbreak clones by mechanisms related to drug purchase and use.

### Acknowledgments

The authors thank Susanne Aebi for performing the PFGE and the staff of the Contact organization, Berne, Switzerland, for supporting the case-control study.

Dr. Böhlen is a specialist in internal medicine with clinical expertise in HIV and infectious diseases. At the time of this study, he was a clinical fellow in the division of infectious diseases of the university hospital of Berne, Switzerland. He is now specializing in dermatology, with a focus on skin infections.

### References

1. Stevens DL. Invasive group A streptococcus infections. Clin Infect Dis 1992;14:2-11.
2. Cleary PP, Kaplan EL, Handley JP, Wlazlo A, Kim MH, Hauser AR, et al. Clonal basis for resurgence of serious *Streptococcus pyogenes* disease in the 1980s. Lancet 1992;339:518-21.
3. Musser JM, Kapur V, Szeto J, Pan X, Swanson DS, Martin DR. Genetic diversity and relationships among *Streptococcus pyogenes* strains expressing serotype M1 protein: recent intercontinental spread of a subclone causing episodes of invasive disease. Infect Immun 1995;63:994-1003.
4. Johnson DR, Stevens DL, Kaplan EL. Epidemiologic analysis of group A streptococcal serotypes associated with severe systemic infections, rheumatic fever, or uncomplicated pharyngitis. J Infect Dis 1992;166:374-82.
5. Schwartz B, Elliott JA, Butler JC, Simon PA, Jameson BL, Welch GE, et al. Clusters of invasive group A streptococcal infections in family, hospital, and nursing home settings. Clin Infect Dis 1992;15:277-84.

6. Kurzynski TA, Van Holten CM. Evaluation of techniques for isolation of group A streptococci from throat cultures. J Clin Microbiol 1981;13:891-4.
7. Burki D, Bernasconi C, Bodmer T, Telenti A. Evaluation of the relatedness of strains of *Mycobacterium avium* using pulsed-field gel electrophoresis. Eur J Clin Microbiol Infect Dis 1995;14:212-7.
8. Beall B, Facklam R, Hoenes T, Schwartz B. Survey of *emm* gene sequences and T-antigen types from systemic *Streptococcus pyogenes* infection isolates collected in San Francisco, California; Atlanta, Georgia; and Connecticut in 1994 and 1995. J Clin Microbiol 1997;35:1231-5.
9. Schwartz B, Facklam RR, Breiman RF. Changing epidemiology of group A streptococcal infection in the USA. Lancet 1990;336:1167-71.
10. Berkelman RL, Martin D, Graham DR, Mowry J, Freisem R, Weber JA, et al. Streptococcal wound infections caused by a vaginal carrier. JAMA 1982;247:2680-2.
11. Rammelkamp CH Jr. Food-borne streptococcal epidemics. N Engl J Med 1969;280:953-4.
12. Summanen PH, Talan DA, Strong C, Mc Teague M, Bennion R, Thompson JE Jr, et al. Bacteriology of skin and soft-tissue infections: comparison of infections in intravenous drug users and individuals with no history of intravenous drug use. Clin Infect Dis 1995;20 Suppl 2:S279-82.
13. Bergstein JM, Baker EJt, Aprahamian C, Schein M, Wittmann DH. Soft tissue abscesses associated with parenteral drug abuse: presentation, microbiology, and treatment. Am Surg 1995;61:1105-8.
14. Navarro VJ, Axelrod PI, Pinover W, Hockfield HS, Kostman JR. A comparison of *Streptococcus pyogenes* (group A streptococcal) bacteremia at an urban and a suburban hospital. The importance of intravenous drug use. Arch Intern Med 1993;153:2679-84.
15. Lentnek AL, Giger O, E OR. Group A beta-hemolytic streptococcal bacteremia and intravenous substance abuse. A growing clinical problem? Arch Intern Med 1990;150:89-93.
16. Passaro DJ, Werner SB, McGee J, Mac Kenzie WR, Vugia DJ. Wound botulism associated with black tar heroin among injecting drug users. JAMA 1998;279:859-63.
17. Clemons KV, Shankland GS, Richardson MD, Stevens DA. Epidemiologic study by DNA typing of a *Candida albicans* outbreak in heroin addicts. J Clin Microbiol 1991;29:205-7.
18. Servant JB, Dutton GN, Ong-Tone L, Barrie T, Davey C. Candidal endophthalmitis in Glaswegian heroin addicts: report of an epidemic. Transactions of the Ophthalmological Society of the United Kingdom 1985;104:297-308.
19. Sundkvist T, Johansson B, Widell A. Rectum carried drugs may spread hepatitis A among drug addicts. Scand J Infect Dis 1985;17:1-4.
20. Sun KO. Outbreak of tetanus among heroin addicts in Hong Kong. J R Soc Med 1994;87:494-5.
21. Hoeger PH, Haupt G, Hoelzle E. Acute multifocal skin necrosis: synergism between invasive streptococcal infection and cocaine-induced tissue ischaemia? Acta Derm Venereol 1996;76:239-41.
